# Evaluation of the use versus nonuse of urinary catheterization during laparoscopic adnexal surgery: A randomized controlled trial

**DOI:** 10.1016/j.heliyon.2024.e27741

**Published:** 2024-03-16

**Authors:** Yujian Jia, Huisheng Ge, Liling Xiong, Lulu Wang, Jieru Peng, Ying Liu, Jie Yu, Jianmei Liao, Hui Wang, Xiaoqin Gan, Yonghong Lin

**Affiliations:** a Department of Gynecology, Chengdu Women's and Children's Central Hospital, School of Medicine, University of Electronic Science and Technology of China, Chengdu, China; b Medical Administrative Department, Chengdu Women's and Children's Central Hospital, School of Medicine, University of Electronic Science and Technology of China, Chengdu, China

**Keywords:** Urinary catheterization, Adnexal surgery, Lower urinary tract symptoms, Microscopic hematuria

## Abstract

We conducted a randomized controlled trial to assess the feasibility and safety of performing gynecological single-port transumbilical laparoscopic-assisted adnexal surgery without urethral catheterization in a day surgery setting. A total of 153 patients with adnexal disease were enrolled in this prospective randomized controlled trial (RCT). All subjects performed single-port transumbilical laparoscopic-assisted adnexal surgery between March 2021 and July 2022 in a day surgery center. After completion of the baseline survey, participants were randomized into one of three groups. Participants were randomized into one of three groups: uncatheterized (n = 51), intermittent catheterized (n = 51), or indwelling catheterized (n = 51). The primary outcomes were the incidence of lower urinary tract symptoms (LUTS) and microscopic hematuria, and the secondary outcomes included the incidence of urinary tract infection (UTI), the incidence of urinary retention, the incidence of bladder injury, the time till first urination, the time till first ambulation, the time till first exhaust, the time till first feeding and Kolcaba comfort score. The incidence of postoperative LUTS in the uncatheterized group (17.65%) was lower than that in the intermittent catheterized group (52.94%) and the indwelling catheterized group (84.31%), and there was significant difference between the two catheterized groups (P < 0.001). In the patients without vaginal manipulation, the incidence of microscopic hematuria in the uncatheterized group (0%) was lower than that in the intermittent catheterized group (37.50%) and the indwelling catheterized group (38.89%) (P < 0.05). There were no significant differences in the first urination time, first ambulation time, first exhaust time, first feeding time, and comfort score among the three groups (P > 0.05). Moreover, no urinary retention, UTI and bladder injury were recorded in the three groups. Gynecological single-port laparoscopic adnexal surgery without urinary catheter is safe and feasible in a day surgery ward, which can reduce the incidence of postoperative LUTS and microscopic hematuria.

## 1 Introduction

Single-port transumbilical laparoscopic-assisted adnexal surgery is safe and feasible and provides almost no visual scar [1]. Day surgery is feasible for women who beneficiate of laparoscopy for adnexal pathology, infertility treatment or exploration [2].

Urinary catheterization was routinely performed before gynecological laparoscopy, without rigorous evaluation. The rationale for catheterization is the belief that an empty bladder has less risk of bladder injury during surgery than a dilated bladder [3]. An inflated bladder can also interfere with exposure and make surgery more difficult. The catheter is either removed at the end of surgery or left in situ for a varying period of time to avoid postoperative urinary retention [4,5]. A recent randomized trial has shown that immediate catheter removal after non-hysterectomy benign gynaecological laparoscopy can increase the risk of urinary retention, and delayed catheter removal does not increase the risk of urinary tract infection (UTI) [6]. However, other researchers have suggested that catheterization is an invasive procedure, which may damage the urethra and bladder mucosa and increase the chance of retrograde bacterial infection. The use of indwelling urinary catheters has been recognized as a major cause of urinary tract contamination [7,8]. Continuation of drainage postoperatively will further increase this risk and delay mobilization by contributing to discomfort. The inappropriate urinary catheter use may increase the associated risk of UTIs, lower urinary tract symptoms (LUTS), catheter-associated pain/discomfort to the woman, and could lead to later ambulation and a longer stay in hospita [9,10]. The risk increases considerably with duration of catheterization [[11], [12], [13]]. In addition, urinary catheterization can also affect the patient's social, work and psychological wellbeing. Thus, most enhanced recovery after surgery (ERAS) guidelines recommended early removal of urinary catheters. The best way to prevent complications is to avoid catheterization whenever possible. Meanwhile, some studies have suggested that no-placement of urinary catheter is safe and feasible in uncomplicated surgery [14,15].

Data regarding the feasibility and safety of the nonuse of urinary catheterization in women undergoing single-port laparoscopic adnexal surgery in a day surgery setting is limited. In this context, given the need to further evaluate the pragmatic use of urinary catheters in patients undergoing gynecological day single-port laparoscopic-assisted adnexal surgery, we decided to perform this prospective randomized controlled trial (RCT).

## 2 Methods

### 2.1 Participants and study settings

The study was conducted at the Gynecological Day Surgery Ward of Chengdu Women's and Children's Central Hospital, Chengdu, China from March 2021 to July 2022. This hospital is a university teaching hospital providing maternity and child care. Eligible women were those undergoing transumbilical single-port laparoscopic-assisted adnexal surgery. All participants could understand the protocol and provided written informed consent. The study was conducted in accordance with the Helsinki Declaration.

Inclusion criteria: age between 18 and 55 years old, unilateral or bilateral adnexal surgery (infertility, hydrosalpinx, tubal anastomosis, or adnexal cyst), American Society of Anesthesiologists (ASA) grade Ⅰ-Ⅱ.

Exclusion criteria: medical complications (such as hypertension, diabetes, heart disease, nephropathy), body temperature > 37.5C, HB < 80 g, suspected malignant tumor, severe pelvic adhesion, other circumstances considered by the investigator to be inappropriate for participation in the trial.

### 2.2 Study design

**Fig. 1:**
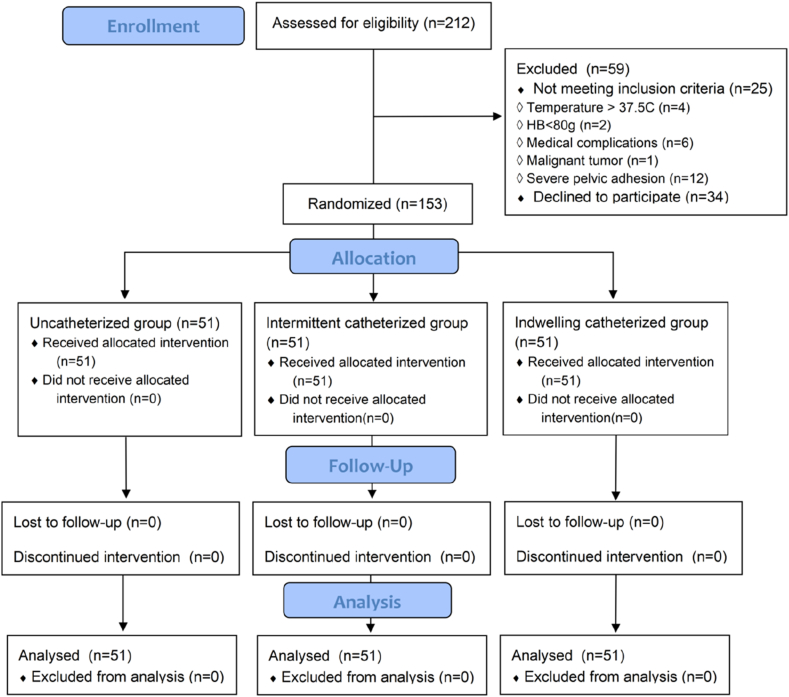
Flowchart of participant enrolment, allocation, and analysis.

As presented in Fig. 1, participants were randomized into one of three groups in a 1:1:1 ratio using opaque, closed envelopes that were mixed and chosen at random. Patients in the uncatheterized group did not place the urinary catheters during surgery. Patients in the intermittent catheterized group were catheterized once after surgery, and the catheters were removed immediately. Patients in the indwelling catheterized group were catheterized during surgery, and the catheters were removed at the end of surgery.

This study was a clinical randomized controlled trial, using single blind method. The participants were given indwelling catheterization or intermittent catheterization after anesthesia. The catheters were removed before anesthesia recovery, so the patient was unaware of whether a urinary catheter was placed during the operation.

### 2.3 Intervention

All laboratory tests were completed, and an informed consent form was signed before surgery. Procedures were started in the standard manner. Within 6 h before surgery, all patients were fasted on dairy products and starchy solid foods (fried, fat and meat foods should be fasted for more than 8 h). The preoperative preparation also encompassed the administration of 250–300 ml carbohydrate-rich drinks up to 2 h before surgery, no skin preparation, no enema, and emptied the bladder 10 min before entering the operating room.

Catheter placement (18Fr double cavity balloon catheter) was determined by the itinerant nurse based on enrollment status after anesthesia. Urine output was recorded in the intermittent catheterized group and the indwelling catheterized group. The surgeon made a 1.5–2.0 cm longitudinal incision in the umbilical region. The transumbilical single incision was made with multifascial trocar placement using conventional laparoscopic instruments. The specimens were bagged and removed through the incision. No negative pressure drainage tube was retained after surgery. All operations were performed by three designated doctors in day surgery ward. General anesthesia was used for the operation.

All patients did not use an analgesic pump. One dose of antibiotics was administered prophylactically within 1 h of incision in order to obtain the highest drug serum levels at incision, and no other liquid infusion was given. Typically, patients could drink a small amount of warm boiled water if there is no dizziness, nausea, vomiting and other discomfort after awakening from anesthesia. If the patients did not feel ill after drinking water, they could eat easily digestible food, except raw, cold, spicy and gas-producing food. In all cases, oral ibuprofen suspension was used for postoperative analgesia. Patients were encouraged to get out of bed as soon as possible after surgery. Two full-time nurses asked and recorded the symptoms of LUTS, the time of first urination, the time of first ambulation, the time of first exhaust and the time till first feeding. The Kolcaba Comfort Scale was used to score comfort status. During the first urination after surgery, clean midstream urine was retained for routine urination test. If there were symptoms of LUTS, and gradually worsening, or accompanied by fever, urine culture should be performed to identify UTI.

### 2.4 Primary and secondary outcomes

The primary outcomes of the study were the incidence of LUTS and microscopic hematuria. LUTS refer to a range of symptoms, including frequency, urgency, burning during micturition, odynuria and suprapubic pain. Microscopic hematuria is defined as ≥ 3 erythrocytes per high magnification field.

The secondary outcomes included the incidence of UTI, the incidence of urinary retention, the incidence of bladder injury, the time till first urination, the time till first ambulation, the time till first exhaust, the time till first feeding and the Kolcaba comfort score. UTI was diagnosed clinically with either the presence of 100 coliform organisms per ml urine with pyuria (≥10 leukocytes per mm^3^) or ≥100 000 of any pathogenic organism per ml urine on culture. Urine retention is defined as the need for at least a single use of intermittent catheterization due to the inability to void adequately.

### 2.5 Sample size considerations

The original power calculation, made prior to the RCT, was based on the data of preliminary study. In brief, the incidence of LUTS in the three groups was about 20%, 50%, and 85%, respectively. The following sample size estimation formula was used: $n=\frac{2\lambda }{{\left(2\;\sin^{-1}\sqrt{p_{\max}}-2\;\sin^{-1}\sqrt{p_{\min}}\right)}^{2}}$. To determine significant differences among the three groups, it was calculated that 13 patients per group were needed (α error, 0.05; β error, 0.1). Anticipating a 20% dropout rate, the total number of patients required for randomization was 16 for each group, giving a total of 48 patients.

### 2.6 Statistical analysis

The results were assessed using the SPSS (Statistical Package for Social Science, version 23.0; IBM) software. Number (n) and percentage (%) were used for categorical variables and mean ± standard deviation for continuous variables. The means of normally distributed continuous variables were compared using one-way ANOVA. If the results of ANOVA was significant, Tukey's test was used to compare the differences between the two means. For categorical data, comparisons were made using the Pearson chi-squared test or Fisher's exact test (and Bonferroni correction). For all analyses a significance level of P < 0.05 was used.

## 3 Results

### 3.1 Basic characteristics

In the present study, a total of 212 patients were screened, and 153 women (51 in each group) were eligible to participate according to the inclusion and exclusion criteria. No significant differences were observed in indices, such as age, BMI, the preoperative hemoglobin levels, the length of the operation, the intraoperative blood loss, the intraoperative infusion volume among the three groups (P > 0.05). There was no statistical difference in the rate of vaginal manipulation (P > 0.05). Further details on demographic data and additional baseline outcome measures are reported in Tables 1 and 2.

**Table 1:** Baseline demographic, clinical and laboratory characteristics of the three groups.

	Uncatheterized group (n = 51)	Intermittent catheterized group (n = 51)	Indwelling catheterized group (n = 51)	*P* value
Age (years)	34.08 ± 7.57	32.75 ± 7.55	33.02 ± 7.69	0.647
BMI (kg/m^2^)	22.01 ± 3.19	22.22 ± 2.94	22.15 ± 3.35	0.945
Preoperative hemoglobin (g/L)	127.59 ± 12.58	125.41 ± 15.14	128.71 ± 11.74	0.444
Operation time (min)	92.18 ± 146.29	85.55 ± 37.55	87.06 ± 48.95	0.929
Intraoperative blood loss (ml)	19.9 ± 28.59	29.45 ± 47.56	21.27 ± 36.85	0.401
Intraoperative infusion volume (ml)	868.63 ± 258.84	956.86 ± 287.23	962.75 ± 353.81	0.216
Intraoperative urine volume (ml)	/	84.73 ± 65.77	85.12 ± 84.12	0.979

**Table 2:** The patients with or without vaginal manipulation in the three groups.

	With vaginal manipulation: n (%)	Without vaginal manipulation: n (%)	*P* value
Uncatheterized group (n = 51)	34 (66.67)	17 (33.33)	0.916
Intermittent catheterized group (n = 51)	35 (68.63)	16 (31.37)	
Indwelling catheterized group (n = 51)	33 (64.71)	18 (35.29)	

### 3.2 Primary and secondary outcomes

The descriptive results of primary outcomes are summarized in Tables 3 and 4. In all patients with or without vaginal manipulation, the results revealed a significant difference in the incidence of LUTS among the three groups (P < 0.001). Pairwise comparisons of the three groups also showed statistical differences. There was no significant difference in the incidence of microscopic hematuria among the three groups (P = 0.651). But in patients without vaginal manipulation, the incidence of microscopic hematuria in the uncatheterized group (0.00%) was lower than that in the intermittent catheterized group (37.50%) and the indwelling catheterized group (38.89%) (P = 0.013), and there was no significant difference between the two catheterized groups. The microscopic hematuria and LUTS of all patients were significantly relieved within two weeks of postoperative follow-up. There were no significant differences in the time till first urination, the time till first ambulation, the time till first exhaust, the time till first feeding and the Kolcaba comfort score among the three groups (P > 0.05) (Table 5). No acute urinary retention, UTI or bladder injury occurred in the three groups.

**Table 3:** Comparison of primary outcomes in the three groups.

	Uncatheterized group (n = 51)	Intermittent catheterized group (n = 51)	Indwelling catheterized group (n = 51)	*P* value
LUTS: n (%)	9 (17.65)	27 (52.94)	43 (84.31)	<0.001
Microscopic hematuria: n (%)	7 (13.73)	10 (19.61)	11 (21.57)	0.651

**Table 4:** Comparison of primary outcomes in the three groups without vaginal manipulation.

	Uncatheterized group (n = 17)	Intermittent catheterized group (n = 16)	Indwelling catheterized group (n = 18)	P value
LUTS: n (%)	0 (0.00)	6 (37.50)	13 (72.22)	<0.001
Microscopic hematuria: n (%)	0 (0.00)	6 (37.50)	7 (38.89)	0.013

**Table 5:** Comparison of secondary outcomes in the three groups.

	Uncatheterized group (n = 51)	Intermittent catheterized group (n = 51)	Indwelling catheterized group (n = 51)	*P* value
Time till first urination (h)	2.14 ± 1.10	2.07 ± 0.97	2.19 ± 0.90	0.822
Time till first ambulation (h)	2.58 ± 1.11	2.50 ± 1.05	2.41 ± 0.97	0.716
Time till first exhaust (h)	3.19 ± 1.54	3.27 ± 1.41	2.71 ± 1.24	0.101
Time till first feeding (h)	7.84 ± 4.51	9.27 ± 4.68	8.75 ± 5.54	0.338
Kolcaba comfort score	86.67 ± 9.64	87.6 ± 8.89	87.84 ± 9.54	0.802

## 4 Discussion

Most patients undergoing major abdominal surgery have a urinary catheter inserted to monitor intraoperative and postoperative urine output. In patients undergoing pelvic surgery, urinary catheters also are used in order to enhance visualization of the field. However, urinary catheters limit early mobility and increase the risk of urinary tract contamination and LUTS [16,17]. In order to reduce the incidence of catheter-associated complications and promote postoperative recovery, the 2019 ERAS Gynecologic/Oncology guidelines recommend that urinary catheters should be used for postoperative bladder drainage for a short period preferably < 24 h postoperatively [18]. With the widespread application of minimally invasive surgery (MIS) and ERAS protocols (such as multimodal analgesia, minimizing perioperative fluid administration, and early resumption of oral intake) in gynecological day surgery, single-port laparoscopic adnexal surgery without urethral catheterization appears to be feasible [[19], [20], [21]].

One of the concerns about the nonuse of catheters is that it might lead to an increased incidence of urinary retention. Recovery of spontaneous micturition is affected after almost any surgical procedure. Postoperative urinary retention occurs if spontaneous voiding delay causes bladder distention beyond the maximum bladder capacity (MBC) [22,23]. If the urinary retention is not treated, overdistention of the bladder wall may occur, which could damage the detrusor muscle and result in a complete inability to urinate, requiring lifelong intermittent self-catheterization. Bladder capacity between 400 and 600 ml is commonly used as a threshold for bladder catheterization to prevent urinary retention [4,23]. In our intermittent catheterized group and indwelling catheterized group, the mean urine volume was 84.73 ml (±65.77 ml) and 85.12 ml (±84.12 ml), respectively. Meanwhile, the maximum volume was less than 400 ml. A slightly filled bladder may be better demarcated and therefore easier to identify intraoperatively. As improvements in surgical and anesthetic technologies, the incidence of urinary retention is relatively low after minor surgical procedures [[24], [25], [26]]. The other advantages of catheterization are prevention of bladder trauma and improved surgical field, which in most patients can be achieved by asking the patient to empty the bladder before entering the operating room. Indeed, urinary retention, bladder injury, and surgical difficulties did not occur in the current study. Thus, we believe that it is feasible to perform gynecological single-site laparoscopic adnexal surgery without urinary catheterization in a day surgery ward.

None of the secondary outcomes in our study achieved statistical significance. The lack of UTIs may be attributed to the brief duration of catheter placement, even among patients with indwelling catheters. It is assumed that LUTS and microscopic hematuria did not occur without the use of urinary catheters, but the incidence of LUTS and microscopic hematuria in the uncatheterized group was 17.65% and 13.73%, respectively. Coincidentally, these symptomatic patients all had vaginal manipulations (hydrotubation or hysteroscopic surgery, curettage, etc.) that may have injured the urethral meatus. As expected, the incidence of LUTS increased in the two catheterized groups. Catheter-related LUTS is caused by involuntary contractions of the bladder, mediated by muscarinic receptors (particularly subtype M3) located in the bladder wall near the catheter [27,28]. The incidence of LUTS in the indwelling catheterized group was higher than that in the intermittent catheterized group. An indwelling catheter ensures the continuous excretion of urine, maintains the empty bladder, enhances the irritation to the bladder wall, and increases the incidence of LUTS [17]. Intermittent catheterization reduces the duration of stimulation of the urinary catheter to the bladder wall.

In patients without vaginal manipulation, the incidence of microscopic hematuria was increased in the two catheterized group, and no patients had microscopic hematuria in the uncatheterized group. The urethral epithelium could be injured by catheterization. We observed no significant differences in the incidence of microscopic hematuria between the two catheterized groups. This may be due to the catheter was removed immediately after surgery, and the duration of catheter placement was relatively short in the indwelling catheterized group. From our observation, it was clear that non-placement of urinary catheters may reduce the incidence of catheter-related urinary complications.

However, some limitations must be considered. One of the limitations is the restricted sample size, which may have decreased the power of the study. Other limitations include inconsistent surgical procedures and no long-term follow-up assessment. We recommend that a larger RCT with a uniform approach and appropriate outcome measures should be performed.

In summary, this study shows that routine urinary catheterization during single-port laparoscopic adnexal surgery is unwarranted. Given the increasing popularity of day surgery, the direct and indirect benefits of avoiding catheterization are likely to be substantial.

## Ethics statement

This study was approved by the Chengdu Women's and Children's Central Hospital Ethics Committee on November 2021. IRB identification number: 2021 (105).

## Funding statement

This study was fund by Chengdu Technological Innovation 10.13039/100006190Research and Development Project (2021-YF05- 00868-SN).

## Data availability statement

Data will be made available on request.

## Clinical trial registry number

Chinese Clinical Trial Registry (ChiCTR); Date of trial registration: January 10, 2021; Identification number: ChiCTR2100041968; URL of the registration site: https://www.chictr.org.cn/showprojen.aspx?proj=120034.

## CRediT authorship contribution statement

**Yujian Jia:** Writing – original draft, Funding acquisition, Data curation. **Huisheng Ge:** Writing – review & editing, Investigation, Data curation. **Liling Xiong:** Data curation. **Lulu Wang:** Investigation, Formal analysis, Data curation. **Jieru Peng:** Formal analysis. **Ying Liu:** Data curation. **Jie Yu:** Data curation. **Jianmei Liao:** Data curation. **Hui Wang:** Data curation. **Xiaoqin Gan:** Writing – review & editing, Data curation. **Yonghong Lin:** Writing – review & editing, Investigation, Data curation.

## Declaration of competing interest

The authors declare that they have no known competing financial interests or personal relationships that could have appeared to influence the work reported in this paper.

## References

[1] Kim T.J., Lee Y.Y., Kim M.J. (2009). Single port access laparoscopic adnexal surgery. J. Minim. Invasive Gynecol..

[2] Bazzurini L., Manfredi G., Roldán E.T. (2022). Same-day discharge protocol for laparoscopic treatment of adnexal disease: management and acceptance. Minim. Invasive Ther. Allied Technol..

[3] Levy B.F., De Guara J., Willson P.D., Soon Y., Kent A., Rockall T.A. (2012). Bladder injuries in emergency/expedited laparoscopic surgery in the absence of previous surgery: a case series. Ann. R. Coll. Surg. Engl..

[4] Baldini G., Bagry H., Aprikian A., Carli F. (2009). Postoperative urinary retention: anesthetic and perioperative considerations. Anesthesiology.

[5] Geller E.J. (2014). Prevention and management of postoperative urinary retention after urogynecologic surgery. Int. J. Womens Health.

[6] McCormack L., Song S., Budden A. (2023). Immediate versus delayed urinary catheter removal following non-hysterectomy benign gynaecological laparoscopy: a randomised trial. BJOG.

[7] Millner R., Becknell B. (2019). Urinary tract infections. Pediatr. Clin..

[8] Rubi H., Mudey G., Kunjalwar R. (2022). Catheter-associated urinary tract infection (CAUTI). Cureus.

[9] Parker V., Giles M., Graham L. (2017). Avoiding inappropriate urinary catheter use and catheter-associated urinary tract infection (CAUTI): a pre-post control intervention study. BMC Health Serv. Res..

[10] Tiwari M.M., Charlton M.E., Anderson J.R., Hermsen E.D., Rupp M.E. (2012). Inappropriate use of urinary catheters: a prospective observational study. Am. J. Infect. Control.

[11] Wald H.L., Ma A., Bratzler D.W., Kramer A.M. (2008). Indwelling urinary catheter use in the postoperative period: analysis of the national surgical infection prevention project data. Arch. Surg..

[12] Trickey A.W., Crosby M.E., Vasaly F., Donovan J., Moynihan J., Reines H.D. (2014). Using NSQIP to investigate SCIP deficiencies in surgical patients with a high risk of developing hospital-associated urinary tract infections. Am. J. Med. Qual..

[13] Okrainec A., Aarts M.A., Conn L.G. (2017). Compliance with urinary catheter removal guidelines leads to improved outcome in enhanced recovery after surgery patients. J. Gastrointest. Surg..

[14] (2011). Is routine indwelling catheterisation of the bladder for caesarean section necessary? A systematic review [published correction appears in BJOG. 2011 May;118(6):774. BJOG.

[15] Deng J., Chen J., Yang T., Guo X., Xie C. (2023). The safety and feasibility of no-placement of urinary catheter after single-port laparoscopic surgery in patients with benign ovarian tumor: a retrospective cohort study. Taiwan J. Obstet. Gynecol..

[16] Flores-Mireles A., Hreha T.N., Hunstad D.A. (2019). Pathophysiology, treatment, and prevention of catheter-associated urinary tract infection. Top. Spinal Cord. Inj. Rehabil..

[17] Wilson M. (2008). Causes and management of indwelling urinary catheter-related pain. Br. J. Nurs..

[18] Nelson G., Bakkum-Gamez J., Kalogera E. (2019). Guidelines for perioperative care in gynecologic/oncology: enhanced Recovery after Surgery (ERAS) Society recommendations-2019 update. Int. J. Gynecol. Cancer.

[19] ACOG Committee Opinion No (2018). 750: perioperative pathways: enhanced recovery after surgery. Obstet. Gynecol..

[20] Zaritsky E., Tucker L.Y., Neugebauer R. (2017). Minimally invasive hysterectomy and power morcellation trends in a west coast integrated health system. Obstet. Gynecol..

[21] Moawad G., Liu E., Song C., Fu A.Z. (2017). Movement to outpatient hysterectomy for benign indications in the United States, 2008-2014. PLoS One.

[22] Joelsson-Alm E., Nyman C.R., Lindholm C., Ulfvarson J., Svensen C. (2009). Perioperative bladder distension: a prospective study. Scand. J. Urol. Nephrol..

[23] Darrah D.M., Griebling T.L., Silverstein J.H. (2009). Postoperative urinary retention. Anesthesiol. Clin..

[24] Tischler E.H., Restrepo C., Oh J., Matthews C.N., Chen A.F., Parvizi J. (2016). Urinary retention is rare after total joint arthroplasty when using opioid-free regional anesthesia. J. Arthroplasty.

[25] Guerra A., Chao C., Wallace G.A., Rodriguez H.E., Eskandari M.K. (2022). Changes in anesthesia can reduce periprocedural urinary retention after EVAR. Ann. Vasc. Surg..

[26] Jackson J., Davies P., Leggett N. (2019). Systematic review of interventions for the prevention and treatment of postoperative urinary retention. BJS Open.

[27] Anderson K.E. (1993). Pharmacology of lower urinary tract smooth muscles and penile erectile tissues. Pharmacol. Rev..

[28] Agarwal A., Raza M., Singhal V. (2005). The efficacy of tolterodine for prevention of catheter-related bladder discomfort: a prospective, randomized, placebo-controlled, double-blind study. Anesth. Analg..

